# The Role of the Mammalian Prion Protein in the Control of Sleep

**DOI:** 10.3390/pathogens6040058

**Published:** 2017-11-17

**Authors:** Amber Roguski, Andrew C. Gill

**Affiliations:** 1 The Roslin Institute and Royal (Dick) School of Veterinary Sciences, University of Edinburgh, Easter Bush Veterinary Centre, Edinburgh EH25 9RG, UK; agproguski@gmail.com; 2 School of Chemistry, Joseph Banks Laboratories, University of Lincoln, Green Lane, Lincoln, Lincolnshire LN6 7DL, UK

**Keywords:** prion, sleep, circadian rhythm, melatonin, serotonin

## Abstract

Sleep disruption is a prevalent clinical feature in many neurodegenerative disorders, including human prion diseases where it can be the defining dysfunction, as in the case of the “eponymous” fatal familial insomnia, or an early-stage symptom as in certain types of Creutzfeldt-Jakob disease. It is important to establish the role of the cellular prion protein (PrP^C^), the key molecule involved in prion pathogenesis, within the sleep-wake system in order to understand fully the mechanisms underlying its contribution to both healthy circadian rhythmicity and sleep dysfunction during disease. Although severe disruption to the circadian rhythm and melatonin release is evident during the pathogenic phases of some prion diseases, untangling whether PrP^C^ plays a role in circadian rhythmicity, as suggested in mice deficient for PrP^C^ expression, is challenging given the lack of basic experimental research. We provide a short review of the small amount of direct literature focused on the role of PrP^C^ in melatonin and circadian rhythm regulation, as well as suggesting mechanisms by which PrP^C^ might exert influence upon noradrenergic and dopaminergic signaling and melatonin synthesis. Future research in this area should focus upon isolating the points of dysfunction within the retino-pineal pathway and further investigate PrP^C^ mediation of pinealocyte GPCR activity.

## 1. Introduction

The highly conserved prion protein (PrP) is encoded by the *PRNP* gene (human cytogenic location 20p12) [1] and exists predominately in two conformationally different isoforms [2]. The cellular prion protein (PrP^C^) has an α-helical structure and is expressed highly within the central and peripheral nervous systems [3] and localized to neuronal and glial cell membranes [4]. PrP^C^ expression has also been identified in tissues beyond the nervous systems, including the intestine, heart and lymph nodes [5]. This widespread distribution of apparently functional PrP^C^, coupled with its highly conserved nature, suggests that it has either one important role or multiple, context-dependent roles throughout the body. Total knockout of PrP^C^ is not associated with major deleterious phenotypes [6], suggesting that the latter hypothesis is more likely to be true. Indeed, PrP^C^ has been implicated in, among other things, immune response modulation [7], myelin maintenance [8], mitochondrial homeostasis [9] and signal transduction as reviewed recently by Castle and Gill [4]. A final possibility is that PrP^C^ does indeed have a single role in a key biochemical pathway that impacts other physiological processes that PrP^C^ is proposed to control; in this possibility, PrP^C^ may be involved only in modulating activity of the top-level system rather than turning it on or off, thereby explaining its apparent redundancy and context-dependent functions. Of relevance to this review is the finding that, in models of PrP^C^ dysfunction or knockout, marked alterations to circadian rhythms occur [10] suggesting a role for PrP^C^ in one of the most essential processes for life: the sleep/wake (or active/rest) cycle.

The prion protein is one of a handful of naturally expressed proteins that can misfold into specific pathogenic isoforms and, hence, become integral to neurodegenerative phenotypes. The prion protein’s conformationally-altered isoform is known as PrP^Sc^, which is enriched in β-sheet structure, is insoluble and which assembles into amyloid plaques and fibrils [11]. The conversion from PrP^C^ to PrP^Sc^ and the subsequent aggregation and oligomerization of PrP^Sc^ results in severe, and ultimately fatal, neurodegenerative prion diseases, also known as transmissible spongiform encephalopathies or TSEs [11,12]. Prion diseases can occur sporadically, due to infection, or as the result of genetic influence [13]. The exact mechanism underlying sporadic conversion from PrP^C^ to PrP^Sc^ is unknown, but it is believed that this mechanism is expedited when PrP^C^ contains a disease-initiating mutation. However, following the initiation of an infection (e.g., by ingesting contaminated meat) exogenous PrP^Sc^ is believed to be used as a template, upon which the functional endogenous PrP^C^ misfolds in a process of autocatalytic conversion [11,12]. The incubation period between inoculation and disease onset is prolonged in prion diseases [11], providing a potential window for neuroprotective interventions [14], but this relies on knowledge of the molecular mechanisms responsible for neuronal loss so that intervention can be targeted effectively and specifically. Identifying such mechanisms is complicated by the fact that different prion disease types (or strains) target pathology to rather different areas of the brain and the information that causes neuropathological targeting is believed to be encoded in the structure of PrP^Sc^. Although multimeric forms of PrP^Sc^ may be toxic to neurons directly, by activating signaling pathways that lead to apoptosis or necrosis, it is also possible that the neuropathology that arises with PrP^Sc^ propagation occurs in combination with loss of function of PrP^C^ [15]. Indeed, during pathogenesis of a prion disease, PrP^C^ expression has been suggested to be downregulated [15,16]. Herein we review whether there may be role for PrP^C^ loss of function in the sleep-wake cycle, a key biological system that is compromised to different extents during various prion diseases.

## 2. Sleep Dysfunction during Prion Disease Pathogenesis

Observations of human prion disease patients provided the first links between PrP^C^/PrP^Sc^ and sleep. In the genetic prion disease fatal familial insomnia (FFI), the “eponymous” clinical feature is severely disturbed sleep, characterized by anxiolytic-resistant insomnia, circadian rhythm dysfunction, sleep fragmentation and altered arousal. Polysomnographic studies show a decrease in total sleep time, decreased REM sleep and loss of REM atonia [17]. Central sleep apneas and decreased slow wave sleep are also common features of FFI clinicopathology [18]. These extensive symptoms are likely to be the result of dysfunction across the various systems regulating sleep. 

To variable degrees, sleep disturbances are also evident in other prion diseases, including various human prion diseases, although they are not currently considered part of the clinical diagnostic criteria. Nevertheless, in a recent study almost 90% of sporadic Creutzfeldt-Jakob disease (spCJD) patients reported sleep dysfunction during clinical evaluation, making sleep disturbance more prevalent than any other diagnostic criteria for CJD [19]. Sleep disturbances were also prevalent clinical complaints in all familial CJD patients examined during a separate study [20]. Certain animal models of prion disease also result in disrupted sleep patterns; rats inoculated with various prion strains have pronounced slow wave sleep decreases [21], rhesus monkeys infected with the human prion disease kuru show complete loss of REM sleep and disrupted sleep stage cycling [22], whilst mice inoculated with the murine prion disease RML show alterations in rest period activity from extremely early in the incubation period [23]. Interestingly, it has been reported that patients with Gerstmann-Sträussler-Sheinker disease (a genetic prion disease characterized predominantly by ataxia and pyramidal dysfunction) do not exhibit sleep alterations [24,25], suggesting that sleep dysfunction is specific to particular prion strains. Given that neuropathology in different prion strains is targeted to different regions of the brain, it follows that molecular or cellular alterations in specific brain regions may underlie some of the prion-induced sleep abnormalities. It is pertinent, therefore, to consider the brain areas that control the different aspects of normal sleep and how these are affected during prion pathogenesis.

The slow wave oscillations of deep, non-REM (NREM sleep) are generated, synchronized and stabilized by the thalamocortical network [26,27], whilst fluctuations in thalamocortical excitability produces the hallmark deflections visualized on EEG traces during NREM sleep: the K-complex and sleep spindle [28]. Cortically-generated K-complexes occur both spontaneously and in response to sensory stimulation, acting to monitor environmental stimuli during reduced states of consciousness and to enhance sleep stability [28]. The K-complex waveform is reflected in thalamic activity, with thalamic neurons reinforcing the K-complex slow oscillation [26]. In response to the K-complex, thalamic reticular neurons generate their own oscillation—known as the sleep spindle—which is thought to be instrumental in thalamocortical plasticity, cognition and memory function [29]. One of the most striking polysomnographic observations in FFI and fCJD patients is a reduction or absence of the thalamocortical K-complex and sleep spindle oscillations [19,30,31,32]. Prion-induced dysregulation of slow wave sleep, sleep instability and the loss of K-complex and sleep spindle oscillations (as well as the memory and cognitive impairments seen in prion diseases) are therefore likely due to the gross neurodegeneration of the thalamocortical network as the disease process reaches the clinical phase [33]. Support for this rationale comes from mouse models of FFI: mice expressing PrP^C^ carrying the D178N mutation (that causes FFI in humans in combination with methionine expressed at codon 129) develop thalamic pathology and exhibit disrupted circadian rhythmicity of sleep and motor activity. Both over-expressing [34] and knock-in [35] FFI transgenic mice are phenotypically similar to human FFI patients, with the overexpressing mice exhibiting abnormal REM-sleep transitioning, loss of sleep spindles, reduced slow wave activity and decreased sleep continuity. Anatomically, these mice also exhibited thalamic degeneration, which, as in human patients, is responsible for the observed breakdown of sleep architecture. However, changes in circadian-regulated motor activity of these mice, including decreased dark-phase activity compared to controls [34], are not readily rationalized by thalamic degeneration and knock-in FFI mice also show significant decreases in dark-phase motor activity compared to controls [35].

Symptoms of insomnia reported by prion disease patients can be explained by the reduction in both sleep stability and sleep maintenance associated with thalamocortical degeneration. It is also likely that the dysregulation of circadian rhythmicity seen in prion disease contributes to symptoms of insomnia. A key organ responsible for the modulation of sleep patterns through synthesis of the circadian hormone melatonin is the pineal gland, and it has been demonstrated that non-prion patients with insomnia exhibit reduced pineal gland volume [36] as well as significantly decreased nocturnal plasma melatonin levels [37]. The pineal gland is a site of high level expression of PrP^C^ [4]. Whilst this may render the pineal gland highly susceptible to infection by prions, it also raises the possibility that reduced levels of functional PrP^C^ during a prion infection [15] may be involved in sleep dysfunction. Thus, the mechanisms of circadian dysfunction evident in prion diseases require further basic science investigation beyond clinical reporting, as currently there is little known of how a prion infection can dysregulate such an essential system. 

## 3. Circadian Rhythm, Homeostatic Sleep Pressure and Melatonin

The sleep-wake cycle is driven by two factors: circadian rhythm and homeostatic sleep pressure [38,39]. Homeostatic sleep pressure is best described as the feeling of sleepiness, such that the longer one goes without sleep, the more tired one becomes. The driving mechanism of homeostatic sleep pressure is thought to be the result of chemical build-up, such as increased levels of adenosine [40], within the brain. Slow wave oscillations during wakefulness and sleep are regulated homeostatically, with slow wave activity decreasing over the sleep period and increasing as time awake increases [41,42]. 

Similar to homeostatic sleep pressure, circadian rhythmicity is the result of endogenous processes, with every cell exhibiting its own intrinsic, oscillatory, circadian rhythm [43]. The oscillations in cellular gene expression, caused by a negative feedback transcription/translation loop, have a natural period of around 24 h, but are then synchronized to the exogenous light/dark cycle [43]. The circadian rhythm is also modulated, to a degree, by other external factors such as exercise and food consumption [44] as well as internal factors including core body temperature and the menstrual cycle [45]. 

The main regulatory hormone of the circadian rhythm is melatonin [39]. Melatonin is part of the tryptophan metabolic pathway [42] and it is synthesized and secreted by both central nervous system (CNS) tissues and organs throughout the body [46]. Within the CNS, melatonin production is entrained to the light/dark cycle, with the hormone acting as a mediator between hypothalamic nuclei and target tissues, relaying diurnal and seasonal timings to the body [47]. As with PrP^C^, the variety of organs capable of melatonin synthesis is suggestive of important, contextual roles for this hormone, including regulation of seasonal and circadian rhythms, reproductive function, modulation of neurotransmission and antioxidant effects [47]. 

**Figure 1:**
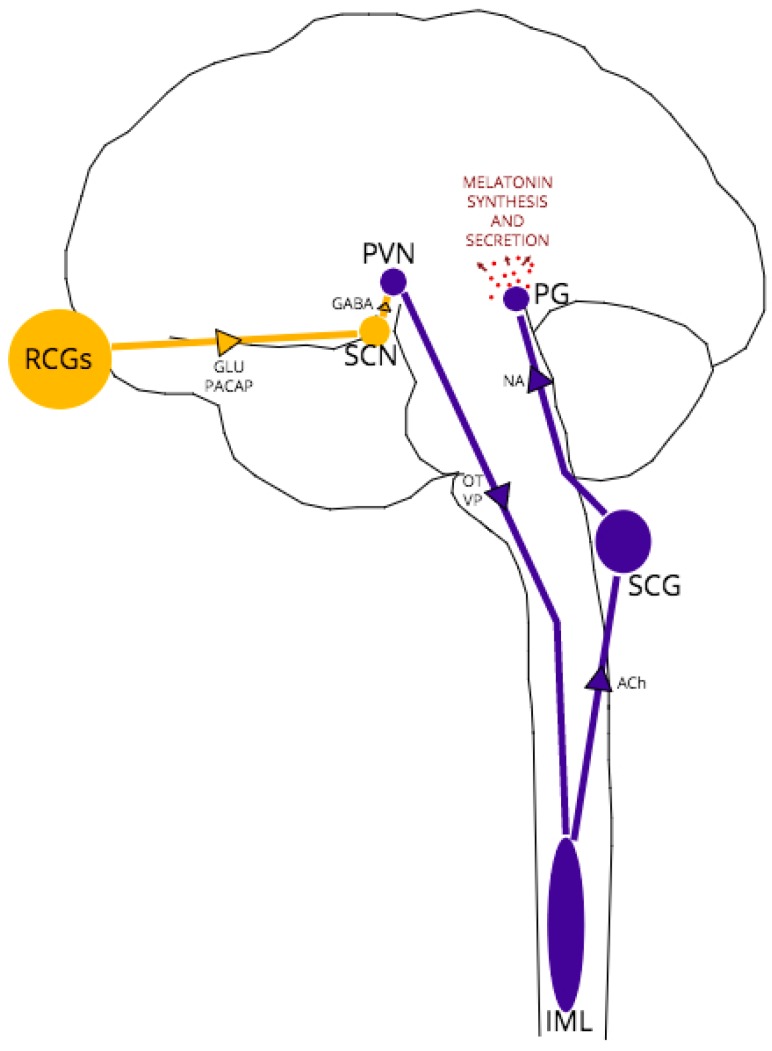
The Retino-Pineal Pathway. Structures and pathways in yellow represent light-active regions, those in dark blue represent dark-active regions. Arrows represent direction of signaling; associated text represents main neurotransmitters in the pathway. Region abbreviations: RCGs *retinal ganglion cells*; SCN *suprachiasmatic nucleus*; PVN *paraventricular nucleus*; IML *intermediolateral nucleus*; SCG *superior cervical ganglion*; PG *pineal gland*. Neurotransmitter abbreviations: GLU *glutamate*; PACAP *pituitary adenylate-cyclase activating peptide*; GABA *gamma-aminobutyric acid*; OT *oxytocin*; VP *vasopressin*; ACh *acetylcholine*; NA *noradrenaline*.

The complex, multi-nuclei pathway for melatonin synthesis, depicted schematically in Figure 1, begins with blue light excitation of photosensitive, melanopsin-containing retinal ganglion cells (RGCs). The RGC axons form the retino-hypothalamic tract which projects to the hypothalamic suprachiasmatic nucleus (SCN), providing glutamatergic excitation of SCN neurons. The SCN is the central pacemaker that fine-tunes the body’s circadian rhythm, conveying the light/dark fluctuations of the external environment to various tissues, including the brain, via oscillatory activity in order to coordinate metabolic function and homeostasis accordingly [48]. These SCN oscillations are generated in response to RCG neurotransmission by a transcriptional-translational feedback loop between clock genes [49].

In all melatonin-synthesizing organisms, light acts as a zeitgeber (environmental cue) for melatonin secretion, with peak melatonin levels arising in the middle of the night’s sleep at approximately 100 times the daytime melatonin level [39]. In daylight, GABAergic SCN projections synapse to paraventricular (PVN) neurons, inhibiting further excitation of the pathway. At night, without RCG/SCN excitation, PVN neurons are disinhibited, leading in turn to excitation of thoracic intermediolateral (IML) neurons and superior cervical ganglia (SCG) neurons. Noradrenergic SCG neurons project to the pineal gland, which expresses both α1 and β1-adrenoceptors [50]. Noradrenaline-adrenoceptor binding then activates adenylate cyclase and the melatonin synthesis cascade within pinealocytes. 

Pinealocytes provide the majority of circulating melatonin, with immediate melatonin release into the blood (bypassing the blood brain barrier) and into CSF circulation at the point of the pineal recess and third ventricle [51]. Melatonin travels via CSF and blood to target tissues expressing G-protein coupled melatonin receptors MT1 and MT2 [52]. MT1 binding has been associated with metabolic regulation, whilst MT2 binding is associated with circadian rhythmicity [46]. Expression of both melatonin receptors is widespread throughout the brain, with localization to neurons of the SCN, thalamus and hippocampus, amongst other structures [41]. 

The pineal gland is not the only source of melatonin; extrapineal melatonin synthesis is thought to occur in a number of organs that express melatonin-synthesizing enzymes, including the heart, liver and placenta [46]. Indeed, the gut expresses melatonin levels 400 times greater than that of the pineal gland, and extrapineal melatonin synthesis has been shown to occur independently of the photoperiod, with an absence of diurnal fluctuations [53]. In contrast to the pineal-synthesized melatonin, which is immediately released into CSF and blood circulation, melatonin synthesized in tissues beyond the CNS is retained within cells [46]. Intracellular melatonin is correlated with its local anti-inflammatory/oxidative effects; in neonates with respiratory distress syndrome, melatonin treatment significantly reduces pro-inflammatory cytokine and nitrate levels [54], whilst melatonin levels in the elderly are inversely correlated with Charlson Comorbidity Index score, indicating a protective role against chronic disease [55].

Abolition of endogenous CNS melatonin has far-reaching consequences for the brain and body; reports of impaired wound healing [56], disrupted circadian organization of the SCN [57], abolition of clock gene expression in adipose tissue [58] as well as altered bone metabolism leading to bone loss [59] provide evidence for the protective function of melatonin in health. The effects of pinealectomy upon sleep are equally as wide-ranging. Long-term changes post-pinealectomy in humans include reduced sleep efficiency and increased switching between sleep stages, indicating a role for melatonin in sleep stability [60]. Pinealectomized rats show a significant decrease in REM-sleep theta power compared to controls [61]. Intriguingly, there are also reports of the loss of hippocampal CA1 and CA3 neurons post pinealectomy in rats [62], which can be reversed by administering exogenous melatonin; since MT1/MT2-expressing hippocampal CA1 and CA3 neurons [63] generate cortical theta oscillations, the theta reduction in pinealectomized rats may be, in part, the result of the hippocampal pyramidal neuron loss. 

Extrapolating the influence of melatonin upon the CNS and body is challenging, given how intertwined the circadian rhythm is with whole-system function. It is important to identify whether prion disease-related sleep/wake dysfunctions are the result of circadian rhythm anomalies caused by PrP^C^ loss of function from the pineal gland, whether anomalies can be explained by neurodegeneration caused by PrP^Sc^-mediated neurotoxicity or whether both affects play a role. This requires a consideration of the link between PrP^C^, circadian rhythms and melatonin signaling.

## 4. A Role for PrP^C^ in the Regulation of Melatonin Synthesis

Early research revealed that PrP mRNA expression peaks during the circadian dark phase at approximately 14 h (zeitgeber time) [64]. This significant increase in PrP mRNA expression precedes melatonin synthesis, with pineal melatonin levels increasing to a peak from 18 to 20 h (zeitgeber time) [65]. Whilst the apparent correlation between PrP^C^ and melatonin levels may be just that—correlative rather than causative—there is some evidence that PrP^C^ levels impact directly or indirectly on melatonin levels and that the high level of PrP^C^ expression in the pineal glands may play a role in regulating melatonin synthesis.

Early studies of PrP knockout (PrP^−/−^) mice highlighted similar sleep disruptions to those reported in human prion disease cases, such as increased sleep fragmentation and altered slow wave activity [10]. It was noted that PrP^−/−^ mice exhibited a significantly longer circadian phase than controls (23.9 h vs. 23.3 h, respectively), as well as demonstrating inverse dark phase activity compared to controls; control mice carried out the majority of their wheel-running during the first half of the dark phase, whilst PrP^−/−^ mice had increased activity towards the end of the dark phase [10]. These findings suggest PrP^−/−^ mice experience a phase shift in their circadian timings, akin to that seen in delayed phase sleep disorders, with circadian period elongation and delay in activity timings [66]. It was also noted that PrP^−/−^ mice exhibited alterations to intrinsic circadian rhythms. Normally, in the complete absence of light, the circadian rhythm deregulates and becomes “free-running”, which manifests in incremental shifts in peak melatonin release from night to day [67], since intrinsic circadian rhythms in mice are slightly greater than 24 h. When placed in complete darkness, control mice exhibited this free-running circadian rhythm, using motor activity as an indicator, whilst, confusingly, the PrP^−/−^ mice maintained 24 h rhythmicity despite no light entrainment [10]. Further to this, PrP^−/−^ mice demonstrate non-cyclical, phase-shifted melatonin release in comparison to controls, with no increase in nighttime plasma melatonin level but increased levels of melatonin during the day relative to controls [68]. 

The above observations beg the question of whether PrP^C^ functions as part of the pathway that turns light cues into melatonin and suggest that disruption of this pathway might be responsible, in part, for circadian abnormalities during prion disease pathogenesis. Progressive circadian disruption is observed in FFI patients, evidenced by decreasing plasma melatonin levels with disease advancement and complete loss of circadian rhythm by end-stage FFI [18,69]. A case study of one FFI patient reported normal function of the SCN, as evidenced by normal core body temperature rhythms and appropriate sleep stage timing, but dissociation of the circadian rhythms for melatonin and cortisol [69]. This suggests normal light/dark entrainment and SCN oscillatory activity, but a “functional interference” somewhere in the retino-pineal tract between the SCN and pineal gland function [69]. 

Reports of circadian phase-shifts and dysregulated melatonin expression in PrP^C^ dysfunction, supported by the above experimental and clinical findings [10,68], suggest a delay in the synthesis or release of melatonin. Establishing the point at which PrP^C^ interacts with the melatonin-synthesis pathway, whether at the pinealocyte or at structure upstream in the circuit, is therefore essential for revealing the mechanism underlying the abnormal circadian cycling. 

As indicated earlier and depicted in Figure 1, functionally, between the SCN and pineal gland lie three structures: the hypothalamic paraventricular nucleus (PVN), the intermediolateral nucleus (IML) of the thoracic spinal column and the superior cervical ganglion (SCG) at the top of the sympathetic chain. The main neurotransmitters released by each structure are vasopressin/oxytocin, acetylcholine and noradrenaline respectively [47]. The fact that melatonin is still synthesized at healthy levels in models of PrP^−/−^ and early-stage TSEs, albeit with an altered expression pattern, suggests that the structures are capable of neurotransmission of reasonable fidelity, but the phasing may be compromised in some way. It has been demonstrated that noradrenaline dysregulation is implicated in TSE pathology; for example, after oral and intraperitoneal prion inoculation, PrP^Sc^ neuroinvasion follows a selective route from the periphery via sympathetic nerves to the brain [70,71]. Noradrenergic cell death [72] and dramatically altered noradrenaline levels in specific brain regions (cerebral cortex, cerebellum, pons) and plasma following intraperitoneal or intracerebral inoculation [71,72,73,74] have also been demonstrated in experimental models of prion disease. However, these effects on noradrenaline expression and neurotransmission have not been demonstrated in PrP^−/−^ models, which suggests the specific targeting of the noradrenergic system is the result of PrP^Sc^ pathology, not a loss of PrP^C^ function in disease. 

**Figure 2:**
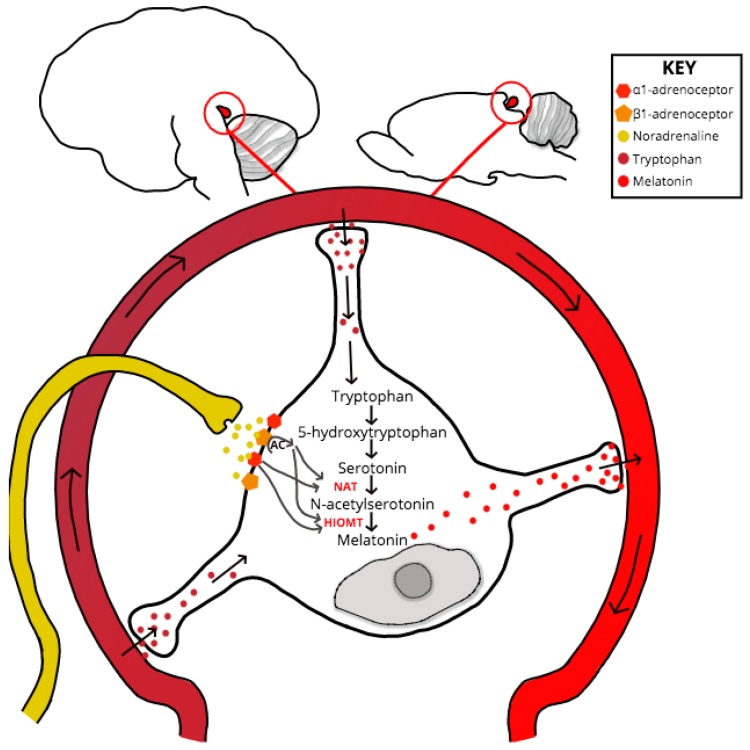
Location of the Pineal Gland in the Human (left) and Mouse (right) Brain and Pinealocyte Melatonin Synthesis. The essential amino acid tryptophan is uptaken by pinealocytes from the surrounding vasculature and is converted to 5-hydroxytryptophan via a process of hydroxylation. Decarboxylation of 5-hydroxytryptophan gives rise to serotonin. SCG afferents, which project to the pineal adjacent to capillaries, release noradrenaline into the pineal perivascular space, where the neurotransmitter binds to α1B and β1-adrenoceptors on the pinealocyte membrane. Coincident activation of adrenoceptors increases cAMP levels, in turn inducing enzyme expression increases of *N*-*acetyltransferase* (*NAT*)—the rate-limiting step of the synthesis pathway- and *acetylserotonin O*-*methyltransferase* (*HIOMT*). Melatonin synthesis is dependent upon serotonin acetylation by *NAT* and *HIOMT* methylation activity of *N*-acetylserotonin. Once synthesized the indolamine is secreted into the circulation, where it has a biological half-life of ~45 min in humans and ~20 min in rats, before hepatic metabolism and urinal metabolite excretion.

If neurotransmission throughout the retino-pineal tract is unchanged in PrP^−/−^, then the point of melatonin dysregulation or synthesis delay must be at the SCG-pinealocyte synapse, or due to a dysfunction at some point downstream and within the melatonin-synthesis signaling pathway (see Figure 2). Despite experimental evidence for PrP mRNA upregulation within the pinealocyte during the subjective night [75], no studies have specifically explored PrP^C^ interactions or co-localization at the pinealocyte. One scenario that might explain the melatonin phase shift exhibited by clinical and experimental models of prion dysfunction is if compromised PrP^C^ function causes dysregulation at the point of serotonin acetylation within the melatonin synthesis pathway. Serotonin is acetylated by the enzyme aralkylamine *N*-acetyltransferase (AA-NAT), whose activity acts as the rate-limiting step of melatonin synthesis [76]. The AA-NAT gene contains a cAMP-responsive element (cre) within its promoter region which, when stimulated by increased levels of cAMP, drives enzymatic expression and activity [77]. Levels of pinealocyte cAMP increase in response to noradrenergic, and to a lesser degree, dopaminergic, binding respectively with adrenergic and D_1_ receptors [77,78].

AA-NAT activity determines melatonin synthesis lag [79], and a significant association between AA-NAT polymorphisms and delayed phase sleep disorder has been shown [76]. Intriguingly, PrP^C^ has continually been proposed to act as a cell-surface scaffolding protein and in this role it may influence AA-NAT activity indirectly through modulating the activity of pinealocyte G protein-coupled receptor (GPCR) complexes. The GPCRs can be divided according to whether they couple with the G_αi_ protein subunit, which inhibits cAMP production and adenylyl cyclase activity, or with the G_αs_ subunit, which inversely stimulates cAMP and adenylyl cyclase activity. Of the main GPCRs expressed by the pinealocyte, only the β-adrenergic and D_1_ GPCRs are G_αs_-coupled [80,81]; it is, therefore, plausible that PrP^C^ associates with one or other of these two GPCRs (or both) as a scaffold protein and influences their signaling cascade in pinealocytes. Supporting this, it has been demonstrated recently that PrP^C^ co-localizes with D_1_ receptors, and there is selective impairment of cAMP signaling in response to D_1_ stimulation in PrP^−/−^ mice [78]. A lack of PrP^C^ may act to reduce adenylyl cyclase activity, resulting in an increased time for cAMP concentrations to reach a supra-threshold level. This would result in dysregulation of AA-NAT, since the length of time taken to achieve optimal acetylation of serotonin increases due to decreased AA-NAT enzymatic activity. Future investigations for a role of PrP^C^ within the circadian rhythm and regulation of melatonin synthesis should therefore investigate protein-protein interactions of PrP^C^ with noradrenergic and dopaminergic receptors, as well as analysis of analytes and catalysts within the melatonin synthesis pathway, with a particular focus on levels of serotonin, *N*-acetylserotonin and AA-NAT.

## 5. Conclusions

Clinical observation and experimental models of prion disease demonstrate clear circadian dysfunction, suggesting a role for PrP^C^ within the synthesis or regulation of melatonin release in health. Drawing upon the limited literature, we propose that PrP^C^ acts as a scaffold protein at the pinealocyte, associating with the G_αs_-coupled β-adrenergic and D_1_ GPCRs. In this hypothesis, PrP^C^ regulates the cAMP signaling cascade downstream of β-adrenergic/D_1_ activation, and in the absence of PrP^C^ (as evidenced by PrP^−/−^ models), a phase shift in melatonin synthesis results due to delayed AA-NAT enzymatic activity.
